# Selections from a Clinical Lecture

**Published:** 1883-09

**Authors:** R. F. Weir

**Affiliations:** Professor of Clinical Surgery in the College of Physicians and Surgeons, New York


### Selections From a Clinical Lecture.

BY R. F. WEIR, M. D.,
Professor of Clinical Surgery in the College of Physicians and Surgeons, New York.

### CARIES OF THE TEETH.

Here is a case which is a very common one, and very often met with in dispensaries. You will be consulted frequently with reference to such cases, for they occur in all classes of life, oftener, however in those who cannot and do not take so good care of their teeth as they should. We find here at the angle of the jaw a little opening in the skin which gives exit to a little pus and blood, and a cord-like swelling running upward and backward toward the inner angle of the jaw. As a rule you will find in these cases one of two conditions on introducing the finger into the mouth; you will find that the cord-like swelling running beneath the skin leads to a hollow tooth, a tooth undergoing decay, or to the alveolar margin where once a decayed tooth existed. In the present instance the trouble lies in the wisdom tooth, as is often true, and has led to the remark that the wisdow tooth is a useless appendage, the last to come and the first to go. You have at once a hint as to the treatment, take out this offending material—the remains of this decayed tooth, which is acting as a foreign body, exciting inflammation; and by so doing you will generally cure your patient. But sometimes the condition is a more complicated one; there has been, first, a carious or dead tooth which acted as a foreign body; this excited inflammation in the alveolar socket; the inflammation extended to the periosteum and involved the bone of the jaw to a imited extent, generally to quite a limited extent, but sufficiently to provoke an abscess which may evacuate externally. Sometimes adhesions form and make a dimple in the cheek from which the patient never recovers. For the relief of this deformity an English surgeon has performed subcutaneous section. I said that in some cases there will be dead bone; but it should be remembered that the bone is sometimes exposed when it is not dead, but is still covered by periosteum, and does not require separation. There is another method in which the wisdom tooth sometimes gives rise to trouble, illustrated in the case of a colored man whom I recently treated. There was a swelling on the side of the face associated with a great deal of neuralgic pain running up over the head. There seemed to be a dead bone at the site of the tumor, but on cutting down upon it the swelling was found to consist of a bony tumor. On cutting through the expanded portion of the jaw I found embedded within it a tooth. This tooth, which was the wisdom tooth, instead of passing upward in a normal manner, forced its way to one side, and caused the condition of things mentioned. It is in this manner that dentigerous cysts are formed, serous fluid accumulating within the walls of the jaw distends them, and forms a tumor with fluid contents. In case during an operation in these cases you have occasion to pull a tooth while the patient is under the influence of ether, you should be careful to grasp the tooth well with the forceps and remove it from the mouth. I failed to do this in one instance ; the patient was taking ether badly, and was coughing considerably, and it was supposed that the tooth had been spat out with the blood. The patient did well immediately afterward, but later the cough became severe, and on careful auscultation Dr. Leaming detected signs of a foreign body within the left bronchus, and I proposed to operate for its removal, but the patient refused, and died in the New York Hospital, the specimen removed from his lung is to be been in the museum to-day. Dr. Leaming had before located a piece of a tracheotomy tube which had become displaced and found its way into the bronchus in the case of a patient under the care of Dr. Guerdon Buck. A forceps was passed down the trachea in that instance, and the obstacle removed.

